# Periventricular Microglia Polarization and Morphological Changes Accompany NLRP3 Inflammasome-Mediated Neuroinflammation after Hypoxic–Ischemic White Matter Damage in Premature Rats

**DOI:** 10.1155/2023/5149306

**Published:** 2023-08-19

**Authors:** Liu Yang, Yajun Zhang, Xuefei Yu, Danni Li, Na Liu, Xindong Xue, Jianhua Fu

**Affiliations:** ^1^Department of Pediatrics, Shengjing Hospital of China Medical University, Shenyang 110004, Liaoning, China; ^2^Department of Pediatrics, The Second Hospital of Dalian Medical University, Dalian 116021, Liaoning, China; ^3^Department of Anesthesiology, Dalian Municipal Maternal and Child Health Care Hospital, Dalian 116021, Liaoning, China

## Abstract

White matter damage (WMD) is a primary cause of cerebral palsy and cognitive impairment in preterm infants, and no effective treatments are available. Microglia are a major component of the innate immune system. When activated, they form typical pro-inflammatory (M1) and anti-inflammatory (M2) phenotypes and regulate myelin development and synapse formation. Therefore, they may play a pivotal role in hypoxic–ischemic (HI) WMD. Herein, we investigated neural inflammation and long-term microglia phenotypic polarization in a neonatal rat model of hypoxia-ischemia-induced WMD and elucidated the underlying pathophysiological processes. We exposed 3-day-old (P3) Sprague−Dawley rats to hypoxia (8% oxygen) for 2.5 hr after unilateral common carotid artery ligation. The activation of NLRP3 inflammatory bodies, microglia M1/M2 polarization, myelination, and synaptic development in our model were monitored 7, 14, and 21 days after birth. In addition, the Morris water maze test was performed on postnatal Day 28. We confirmed myelination disturbance in the periventricular white matter, abnormal synaptic development, and behavioral changes in the periventricular area during the development of HI WMD. In addition, we found an association between the occurrence and development of HI WMD and activation of the NLRP3 inflammasome, microglial M1/M2 polarization, and the release of inflammatory factors. NLRP3 inhibition can play an anti-inflammatory role by inhibiting the differentiation of microglia into the M1 phenotype, thereby improving myelination and synapse formation. In conclusion, microglia are key mediators of the inflammatory response and exhibit continuous phenotypic polarization 7–21 days after HI-induced WMD. This finding can potentially lead to a new treatment regimen targeting the phenotypic polarization of microglia early after HI-induced brain injury.

## 1. Introduction

With advancements in neonatal rescue technology, the survival rate of critical preterm infants has considerably improved. However, consequently, the incidence of brain injury and neonatal sequelae has also increased. Approximately 10% of preterm infants with a gestational age of 33 weeks or less will have cerebral palsy, and approximately 35% will have persistent cognitive and neuropsychiatric defects [1–3]. Premature infants are more vulnerable to various perinatal environment stressors, which could result in injuries to various organs, particularly the brain, than infants born at full term due to their low-gestational age and incomplete organ development. Periventricular white matter damage (WMD) is a common type of brain injury in preterm infants and is the leading cause of cerebral palsy and cognitive impairment. The neuropathological features of WMD primarily include cerebral white matter ischemia, coagulative necrosis, myelination impairment, microglia activation, astrocyte proliferation, and neuronal death [4], which eventually lead to cerebral palsy and disorders of cognition, language, and behavior, among others [5, 6]. The clinical manifestations of WMD in preterm infants are often atypical, and diagnosis is often delayed. Even after diagnosis, effective neuroprotective strategies are lacking. Therefore, to reduce the medical and societal burden associated with WMD, there is an urgent need to explore its pathogenesis and develop effective therapeutic strategies.

WMD in preterm infants occurs due to several factors and is often caused by cerebral ischemia, hypoxia, and injuries caused by inflammatory reactions. Current studies suggest that the pathogenesis of WMD includes inflammatory responses, oxidative stress, free radical injury, cytokine toxicity, and glutamate-evoked excitotoxic injury [7]. Among them, neuroinflammation plays a central role in the development of neonatal brain injury [8], and neonatal hypoxia–ischemia can trigger the inflammatory response [9]. Previous studies have suggested that systemic inflammation induced by HI can affect oligodendrocyte maturation through neuroinflammatory processes, including microglial activation [8, 10]. Furthermore, activated microglia can continuously secrete pro-inflammatory factors, such as tumor necrosis factor (TNF)-*α*, interleukin (IL)-1*β*, IL-6, and components of complementary pathways, resulting in WMD after HI injury [11]. Therefore, reducing neuroinflammation is considered an effective strategy for treating neonatal WMD.

Neuroinflammation can be activated by members of the nucleotide-binding oligomerization domain- (NOD-) like receptor (NLR) family. Among them, the NLR family pyrin domain containing 3 (NLRP3) inflammatory corpuscles play a fundamental and widespread role in the inflammatory response [12, 13]. NLRP3 inflammasome activation is a major driver of neuroinflammation [14] and has been proven to promote pro-inflammatory cytokine secretion and subsequent inflammatory responses [15–17]. In addition, research has shown that when NLRP3 inflammasomes are suppressed, HI brain damage can be alleviated [18]. Microglia are the primary expression site for NLRP3 inflammatory bodies, thus playing a key role in neuroinflammation [19]. Microglia can be activated through various signaling pathways and differentiate into pro-inflammatory (M1) and anti-inflammatory (M2) phenotypes, from which, M1 or M2 mediators are released, which aggravate or promote brain injury or facilitate repair, respectively [20, 21]. Previous studies have shown close relationships between microglia and immature oligodendrocytes, other glia, and vascular endothelial cells [22]. After neonatal HI injury, the number of M2 microglia decreases while the number of M1 microglia increases. Inhibiting M1 microglial activation or transforming M1 microglia into the M2 phenotype can reduce WMD and improve cognitive function [23, 24]. Research has shown that microglial maturation is associated with changes in morphology and gene expression and that microglial responses to ischemia in the developing brain differ based on the age at which injury occurs [25]. As described above, HI-induced neuroinflammation could be one of the main mechanisms mediating the pathological changes leading to WMD in preterm infants. As such, there is strong interest in the development and use of pharmacological inhibitors of microglial activation to limit their proinflammatory and cytotoxic mechanisms. Culminating evidence has shown that selective inhibition of the NLRP3 inflammasome can effectively reduce the expression of pro-inflammatory cytokines (such as IL-1*β* and IL-6) and increase the expression of anti-inflammatory cytokines (such as IL-10 and TGF-*β*) in a model of intracerebral hemorrhage induced by autologous blood and bacterial collagenase [26]. The above results indicate that the microglial phenotype changed to an anti-inflammatory status when the NLRP3 inflammasome was inhibited. Therefore, we have reason to hypothesize that activation of the NLRP3 inflammasome may promote microglial polarization toward the M1 phenotype, thus aggravating neuroinflammation, and the application of selective NLRP3 inflammasome inhibitors will accelerate the M1 to M2 phenotypic shift. Recent research has shown that melatonin could inhibit the hyperactivity of NLRP3 inflammasomes by enhancing mitochondrial autophagy and inhibiting TLR4/NF-*κ*B pathway activity [27]. Minocycline treatment, an antibiotic which limits microglial activity, completely abolished the postnatal Day 3 (P3) HI-induced increase in numbers of activated microglia in the corpus callosum (CC) on postnatal Day 10 (P10) [28]. In addition, our previous study found that caffeine can improve WMD by inhibiting the activation of NLRP3 inflammasomes and correcting the microglial polarization imbalance [29], thereby identifying its key role in improving WMD through proteomics and confirming its association with neuroinflammation [30]. Through the above research, we found that the neuroprotective effects of these drugs have may be related to the broad-spectrum inhibition of the different microglial phenotypes (e.g., inhibiting both toxic and neuroprotective phenotypes). Therefore, therapeutic interventions that specifically block neurotoxic microglial phenotypes may have the greatest potential to protect the injured brain. This study aimed to investigate WMD-related changes in myelin sheaths, synapses, and microglia activation in the periventricular white matter of neonatal rats and the role of NLRP3 in combating neuroinflammation in white matter injury in premature infants, thus proposing potential treatment strategies.

## 2. Materials and Methods

### 2.1. Animals and Ethical Permission

All animal experiments were approved by the Animal Ethical Committee of China Medical University, Shenyang, China (2021PS839K). Perinatal Sprague−Dawley (SD) rats were purchased from Liaoning Changsheng Biotechnology Co., Ltd. (Liaoning, China). All rats were kept with nursing mothers and housed at 25 ± 5°C and 50% humidity in a 12/12 hr light/dark cycle facility with food and water provided ad libitum. All efforts were made to reduce the number of animals used and their suffering. The litter sizes were of 11 or 12 pups. Animals from each litter were randomly allocated to each experimental group.

### 2.2. Hypoxia–Ischemia-Induced Model of Cerebral WMD in Neonatal Rats

Based on previously reported procedures [31, 32], a neonatal rat model of HI-induced cerebral WMD was prepared. The rats were randomly divided into three groups: sham, model (HI), and MCC950 treatment (MCC950). In brief, the 3-day-old male and female SD rats were anesthetized using 3%–5% isoflurane (26675-46-7, Abbott Laboratories, Switzerland) inhalation and 1.5%–2.0% for maintenance, and were fixed on the operating table in a supine position; the left common carotid artery was subsequently exposed under a dissecting microscope (SZ-51, Olympus Corporation, Tokyo, Japan). Permanent ligation was performed on the HI group using sterile needle sutures (6-0, R611, Jinhuan Medical, China). At both ends of the artery, the blood vessel was cut in the middle of the two ligature points, and the wound was sutured. The operation time was 8−10 min. After the operation, the rat was awakened and returned to its mother to recover for 1 hr before being placed in a gas-tight hypoxic box that was continuously supplied with a gas mixture of 8% O_2_ and 92% N_2_ for 2.5 hr at a flow rate of 2 L/min. In all procedures, the body temperature of the rats was maintained between 36 and 37°C using a heating pad (V-100, Yuyan Technology Corporation, Shanghai, China). No deaths occurred during the operation and from postnatal Day 4 (P4) to P10.

**Figure 1:**
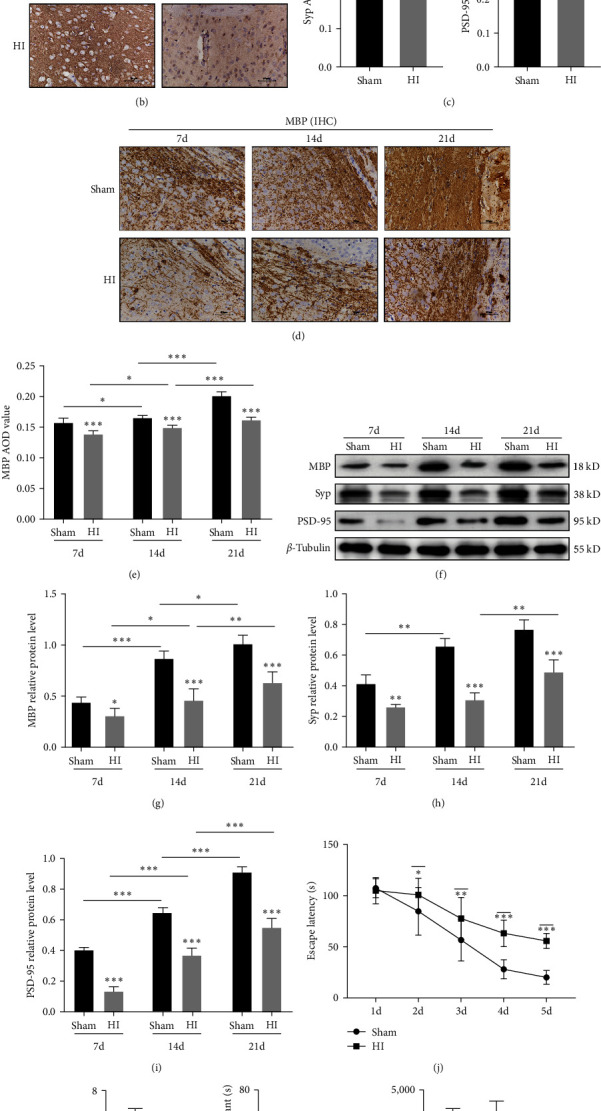
Disorders of myelin sheath development and synaptic formation in the periventricular white matter and decline of long-term learning and cognitive impairment in neonatal rats with hypoxic–ischemic- (HI-) induced white matter damage (WMD). (a) Schematic diagram of the experimental design. (b) IHC staining showing synaptophysin (Syp) and PSD-95 at 21 days after HI injury. Scale bar = 50 *μ*m. (c) AOD value of Syp and PSD-95. (d) IHC staining showing MBP in the corpus callosum (CC) 7, 14, and 21 days after HI injury. Scale bar = 50 *μ*m. (e) AOD value of MBP. (f) Western blot detection of MBP, Syp, and PSD-95 levels. Analysis of relative (g) MBP, (h) Syp, and (i) PSD-95 levels, with *β*-tubulin used for normalization. (j) Escape latencies of rats in the training trials for the hidden platform task. (k) Frequency of platform crossing (times). (l) Time spent (s) in the target quadrant during the probe trial. (m) Moving distance (cm) in the probe trial. Data are presented as mean ± SEM. Statistical analyses were performed using two-way ANOVA, followed by Tukey's test.  ^*∗*^: Sham vs. HI, or comparison of the same group at different times.  ^*∗*^*P* < 0.05,  ^*∗∗*^*P* < 0.01, and  ^*∗∗∗*^*P* < 0.001. Sham group (*n* = 6); HI group (*n* = 6).

The rats in the sham operation group had their left common carotid artery stripped without receiving ligation and hypoxia treatment. MCC950 is an efficient and highly selective inhibitor of NLRP3 inflammasomes [33, 34]. The MCC950 group received additional intraperitoneal injections of MCC950 (20 mg/kg dissolved in 0.9% normal saline) [35] 1 hr before establishing HI-induced cerebral WMD. Whereas the sham and HI groups received an equal volume (0.9%) of intraperitoneally injected normal saline 1 hr before model establishment (Figures 1(a)). The MCC950 was produced by Sigma–Aldrich (C141; Lyon, France). The specimens were collected 7 (P10), 14 (P17), and 21 days (P24) after model establishment (specimens from the MCC950 group were only collected on P17). The rats were euthanized with sodium pentobarbitone (80 mg/kg, intraperitoneal injection). Subsequently, the chest was incised and the heart was exposed. In one group, the rats were perfused with normal saline, and the brain tissue of the lateral cerebral hemisphere was separated on ice and stored in EP tubes (1.5 mL, MCT-150-C, Axygen, USA) at –80°C. In another group, the rats were perfused with 4% paraformaldehyde (80096618, National Pharmaceutical Group, China) after anesthesia, and the brain tissue was extracted and placed in 4% paraformaldehyde for follow-up experiments. Ten rats from each group were randomly selected at each time point for inclusion in different experiments, and histology on paraffin-embedded sections, western blot, polymerase chain reaction (PCR), and enzyme-linked immunosorbent assay (ELISA) analyses were performed. Finally, 6 of the 10 randomly selected rats from each group were included in the statistical analysis.

### 2.3. Morris Water Maze (MVM) Test

At 28–33 days following model development, MWM experiments were conducted [31]. The experiments were performed using a circular pool (diameter: 160 cm and height: 60 cm), containing a movable platform (diameter: 12 cm and height: 28.5 cm) and black inner walls. The pool wall was marked in the middle parts of the four quadrants. Subsequently, we filled the pool with water to a depth of 30 cm, and the height of the platform was 1.5 cm below the water's surface. The temperature was maintained at 25°C throughout the experiment. The test was performed in two phases: the probe trial and acquisition training. The training phase was performed for five consecutive days and there were four training sessions per day where each rat was allowed 120 s to find the platform. For each session, the training stopped when the rat found the submerged platform and stayed on it for 5 s. We recorded the time needed for the rat to find the platform as the escape latency. The swimming distance covered by the rat was also recorded using the system software. Rats that failed to find the platform within 120 s were guided onto the platform and allowed to rest for 20 s. In such cases, the escape latency was recorded as 120 s. On Day 6, we removed the platform and performed the probe trial. In brief, rats were positioned on the platform in the opposite quadrant and were allowed to swim for 120 s. A video tracking system (Shanghai Mobile Datum Ltd., Shanghai, China) was employed to record data. All investigators were blinded to the experimental groups. Several variables of the MWM test, such as moving distance (cm), frequency of platform crossing (times), time spent in the target quadrant (s), and escape latency, were recorded to assess the impact of HI on cognitive impairment. Six rats from each group were selected for the MWM test. After the experiment, all the rats used were euthanized by cervical dislocation.

### 2.4. Immunohistochemical (IHC) Analysis

Brain tissues were sectioned and baked. This was followed by dewaxing with xylene (10023418, National Pharmaceutical Group, China) and hydration using gradient concentrations of ethanol solution (10009218, 100%, 95%, 85%, and 75%, National Pharmaceutical Group, China). They were then heated for 30 min in citrate buffer (10007118, pH 6.0; National Pharmaceutical Group, China) to achieve antigen retrieval. IHC was performed using the Immunohistochemical Hypersensitivity UltraSensitive™ SP Kit (KIT-9710, Maixin Biotechnologles, China). The sections were incubated with hydrogen peroxide (3%) for 20 min and blocked with goat serum for 30 min. After an overnight incubation at 4°C with rabbit antimyelin basic protein (MBP) monoclonal antibody (mAb; 1 : 5,000; ab218011; Abcam, Cambridge, UK), rabbit anti-PSD-95 polyclonal antibody (pAb; 1 : 250; 20665-1-AP; Proteintech, Rosemont, IL, USA), rabbit antiallograft inflammatory factor 1 (AIF1)/ionized calcium-binding adaptor molecule 1 (Iba-1) pAb (1 : 100; Cat.# DF6442; Affinity, Jiangsu, China), and rabbit antisynaptophysin (syp) pAb (1 : 1,000, 17785-1-AP, Proteintech), the sections were heated and incubated with their respective streptavidin–horseradish peroxidase (HRP)-conjugated secondary antibodies at 37°C for 20 min. The samples were stained with 3,3′-diaminobenzidine, redyed, dehydrated, transparentized, and sealed. Finally, a light microscope (Olympus Corporation, Tokyo, Japan) was utilized to observe the sections that were then processed using ImageJ software (NIH, Bethesda, MD, USA). The positive signals detected were quantified based on a predetermined threshold to obtain an average optical density (AOD) value.

### 2.5. Immunofluorescence

Tissue sections were incubated with goat serum (SP KIT-B1, Maixin Biotechnologles, China) at 37°C for 30 min after deparaffinization and antigen retrieval. They were then incubated overnight at 4°C with rabbit anti-NLRP3 pAb (1 : 200; ab214185; Abcam), rabbit anti-CD206 pAb (1 : 500; ab125028; Abcam), rabbit anti-CD86 pAb (1 : 200; 13395-1-AP; Proteintech), and mouse anti-Iba-1 (1 : 100; ab15690; Abcam). This was followed by heating and incubation with an Alexa Fluor 594-conjugated (1 : 200; ab150116/ab150080; Abcam) and Alexa Fluor 488-conjugated (1 : 200; ab150113/ab150077; Abcam) secondary antibody at 24–26°C for 4 hr. Next, we counterstained the tissues with 4′,6-diamidino-2-phenylindole and used a confocal laser-scanning microscope (C1; Nikon, Tokyo, Japan) for imaging. Finally, the average fluorescence intensity of NLRP3 was determined using ImageJ software (NIH).

### 2.6. Western Blotting

On Days 7, 14, and 21 after HI injury, we euthanized the rats and harvested the brains. This was followed by stripping of the cortex and isolation of brain tissues from the periventricular areas on the ligated side. The samples were handled on ice and stored at −80°C and were later processed for western blotting. Proteins were electrophorized using the Omni-Easy™ One-Step PAGE Gel Fast Preparation Kit (PG211/PG212/PG213, Shanghai Epizyme Biomedical Technology Co, China). The following antibodies were used: rabbit anti-MBP (1 : 1,000, Abcam), rabbit anti-AIF1/Iba-1 (1 : 1,000; Affinity), rabbit anti-CD86 (1 : 1,000; Proteintech), rabbit anti-CD206 (1 : 1,000; Abcam), rabbit anti-iNOS pAb (1 : 1,000; 18985-1-AP; Proteintech), rabbit anti-Arginase-1 pAb (1 : 5,000; 16001-1-AP; Proteintech), rabbit anti-NLRP3 (1 : 1,000; Abcam), rabbit anticaspase-1 mAb (1 : 1,000; ab207802; Abcam), and rabbit anti-IL-1*β* pAb (1 : 1,000; Cat.#:AF5103; Affinity). Rabbit anti-*β*-tubulin pAb (1 : 5,000; 10068-1-AP; Proteintech) was used as a loading control. The PVDF membranes (IPVH00010, Millipore, USA) were washed and incubated with an HRP-conjugated goat antirabbit secondary antibody (1 : 5,000; SA00001-2; Proteintech) and then developed using enhanced chemiluminescence reagents (Thermo Fisher Scientific, Waltham, MA, USA). The relative protein band intensity was examined using ImageJ software (NIH). The expression of target proteins was normalized to that of *β*-tubulin.

### 2.7. Real-Time PCR

The reverse transcription–polymerase chain reaction (RT–PCR) technology was employed to assess the mRNA expression levels of transforming growth factor TGF-*β*, IL-10, TNF-*α*, and IL-1*β*. Specimens from the left midbrain region were treated with the TRIzol kit (Takara Bio, Dalian, China) reagents to extract RNA, which was used to synthesize cDNA using HiScript QRT SuperMix for quantitative PCR (qPCR) (+gDNA wiper; R123-01; Vazyme Biotech Co., Ltd., Nanjing, China). The ChamQ universal SYBR qPCR master mix (Q711; Vazyme Biotech Co., Ltd.) was employed to run the RT–PCR reactions. The relative expression level of target mRNA was calculated using the 2^−*ΔΔ*^CT method, with *Gapdh* serving as the reference gene. Primers were generated by the Shanghai Biotechnology Service Co. (Shanghai, China) and had the following sequences:

 
*Il1b* forward 5′-AATCTCACAGCAGCATCTCGACAAG-3′ and reverse 5′-TCCACGGGCAAGACATAGGTAGC-3;
 
*Tnfa* forward, 5′-GCATGATCCGAGATGTGGAACTGG-3′ and reverse 5′-CGCCACGAGCAGGAATGAGAAG-3′;
 
*Il10* forward, 5′-CAAGGCAGTGGAGCAGGTGA-3′ and reverse, 5′-CCGGGTGGTTCAATTTTTCATT-3′;
 
*Tgfb* forward 5′-GACCGCAACAACGCAATCTATGAC-3′ and reverse 5′-CTGGCACTGCTTCCCGAATGTC-3;
 
*Gapdh* forward: 5′-GACATGCCGCCTGGAGAAAC-3′ and reverse 5′-AGCCCAGGATGCCCTTTAGT-3′.

### 2.8. Enzyme-Linked Immunosorbent Assay (ELISA)

The levels of inflammatory factors in the periventricular tissue on the ligated side were determined using ELISA. First, the phosphate-buffered saline solution was added to the brain tissue, which was then homogenized and centrifuged for 15 min at 3,500 rpm to obtain the supernatant. IL-1*β* (EK0393; Boster, Wuhan, China), TNF-*α* (EK0526; Boster), IL-10 (EK0418; Boster), and TGF-*β* (EK0514; Boster) levels were then detected using ELISA by measuring the optical density at 450 nm using a microplate reader (Multiskan FC; Thermo Fisher Scientific).

### 2.9. Imaging and Analysis

The immune-stained sections were observed under an Olympus BX51TF microscope (Melville, NY, USA). Images were captured from the periventricular white matter ranging from 1.5 mm before the bregma to 0.5 mm after the bregma. The detection areas were mainly the CC and the subventricular zone (SVZ). We randomly selected three microglia cells from each of these areas from every rat and performed Sholl analysis for the morphological evaluation of the microglial cells. The immunoreactivity of the cells was quantified using the ImageJ software package (NIH).

### 2.10. Statistical Analyses

Data are presented as the mean ± standard error of the mean. The Kolmogorov–Smirnov test with Dallal–Wilkinson–Lillie correction for *P*-values was performed for normality assessment. Data on escape latency or time were analyzed using a two-way repeated-measures analysis of variance (ANOVA). A parametric one-way ANOVA with Tukey's post hoc test was used to compare all other results. GraphPad Prism software v.8.01 (GraphPad Software, La Jolla, CA, USA) was used for the analyses, and statistical significance was set at *P* < 0.05.

## 3. Results

### 3.1. Disorders of Myelin Sheath Development and Synaptic Formation in Periventricular White Matter and Decline of Long-Term Learning and Cognitive Impairment in Neonatal Rats with HI-Induced WMD

Neonatal hypoxic–ischemic-induced WMD often damages in the SVZ and CC area and often results in myelin and synaptic dysplasia. We evaluated synaptic formation using the presynaptic protein synaptophysin and postsynaptic protein PSD-95. Immunohistochemical analysis results showed that synaptophysin in the periventricular area in the sham group was granular and diffuse at 21 days after HI. In contrast, the synaptophysin level in the periventricular area in the HI group decreased (*P* < 0.01). Similarly, the expression of PSD-95 in the periventricular area was lower in the HI group than in the sham group at 21 days after HI (*P* < 0.01; Figures 1(b) and 1(c)). Western blotting showed that the synaptophysin level in the ligated periventricular area in the sham group increased from 7 to 14 days after HI (*P* < 0.01); however, in the HI group, it increased from 14 to 21 days (*P* < 0.01). The PSD-95 level increased continuously from 7 to 21 days in both the HI and sham groups (all *P* < 0.001). In the HI group, synaptophysin and PSD-95 expression showed a trend for reduced levels (Figures 1(f), 1(h), and 1(i)). The expression of synaptophysin was lower in the HI group than in the sham group at Days 7, 14, and 21 after HI (*P* < 0.01, *P* < 0.001, and *P* < 0.001, respectively; Figures 1(f) and 1(h)). Similarly, the expression of PSD-95 in the HI and sham groups followed this trend at Days 7, 14, and 21 after HI (all *P* < 0.001; Figures 1(f) and 1(i)). The continuous decrease in presynaptic protein synaptophysin and postsynaptic protein PSD-95 levels suggested synaptic formation disorder of the periventricular white matter after HI injury.

MBP is an important protein involved in myelination in the central nervous system, and is expressed throughout the myelin sheath [36, 37]. We used the myelin protein MBP as a marker for myelin development. Immunohistochemical analysis revealed that the MBP level in both the sham and HI groups exhibited a tendency to increase at 7, 14, and 21 days after HI (Figures 1(d) and 1(e)). Compared with the sham group, MBP and myelinated nerve fiber densities significantly decreased in the ligated periventricular CC area in the HI group at each time point (all *P* < 0.001; Figures 1(d) and 1(e)). Western blotting revealed that the MBP level in the periventricular area in the sham group increased continuously from 7 to 21 days after model establishment. In the HI group, MBP expression showed a trend for reduced levels (Figures 1(f) and 1(g)). Compared with the sham group, the expression of MBP in the periventricular area in the HI group decreased significantly at 7, 14, and 21 days after HI (*P* < 0.05 or *P* < 0.001; Figures 1(f) and 1(g)). The MBP level in the HI group decreased continuously, suggesting the presence of myelin development disorder in the periventricular white matter after HI injury.

The MWM test was conducted 28 days after model establishment. In the positioning navigation test, the escape latency of the rats in the HI group from Days 2 to 5 in the training phase was more prolonged than that in the sham group (*P* < 0.05, *P* < 0.01, *P* < 0.001, and *P* < 0.001, respectively; Figure 1(j)). In the space exploration test, the number of times a rat crossed the platform was reduced in the HI group compared to the sham group (*P* < 0.01; Figure 1(k)), and the time spent swimming in the target quadrant was shortened (*P* < 0.01) (Figure 1(l)). In addition, swim distance results to assess athletic ability showed that the observed differences between the groups were not caused by differences in athletic ability (Figure 1(m)). These results suggest that the learning and cognitive ability of the rats declined after HI injury.

### 3.2. Activation of NLRP3 Inflammasomes in the Periventricular White Matter in Neonatal Rats with HI-Induced WMD

**Figure 2:**
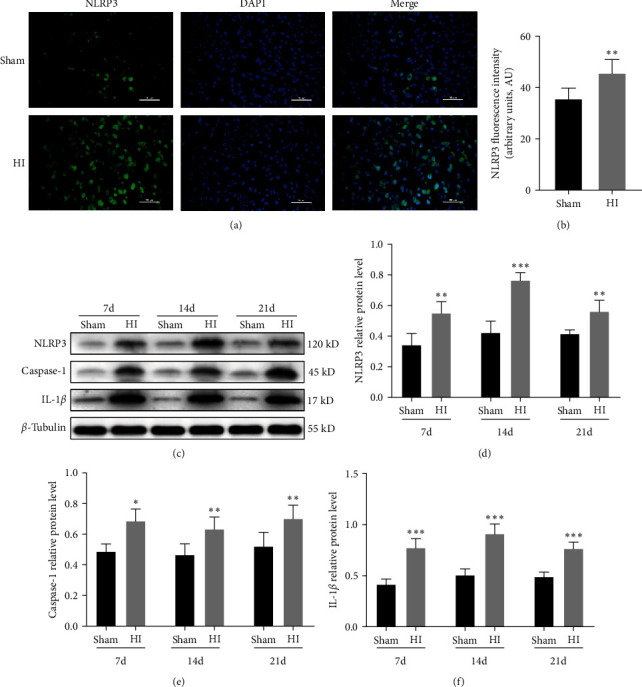
Activation of the NLRP3 inflammasome in the periventricular white matter in neonatal rats with hypoxic–ischemic (HI)-induced white matter damage (WMD). (a) Immunofluorescence staining and (b) fluorescence intensity analysis of NLRP3. Scale bar = 50 *μ*m. (c) Western blot detection of NLRP3, caspase-1, and IL-1*β* levels. Analyses of relative (d) NLRP3, (e) caspase-1, and (f) IL-1*β* levels, with *β*-tubulin used for normalization. Data are presented as mean ± SEM. Statistical analyses were performed using two-way ANOVA, followed by Tukey's test.  ^*∗*^*P* < 0.05,  ^*∗∗*^*P* < 0.01, and  ^*∗∗∗*^*P* < 0.001. Sham group (*n* = 6); HI group (*n* = 6).

Immunofluorescence results revealed that on Day 14 after HI, the fluorescence intensity of NLRP3 in the ligated side of the periventricular white matter area increased semiquantitatively in the HI group, compared to the sham group (*P* < 0.01; Figures 2(a) and 2(b)). In addition, western blotting showed a greater increase in the protein levels of NLRP3, caspase-1, and IL-1*β* in the ligated side of the periventricular white matter area at Days 7, 14, and 21 after HI in the HI group than in the sham group (*P* < 0.05, *P* < 0.01, and *P* < 0.001, respectively; Figure 2(c)–2(f)). These results suggest that the NLRP3 inflammasome is activated in the periventricular white matter after HI injury.

### 3.3. Increased Number and Changes in Microglial Iba-1 Morphology in the Periventricular White Matter in Neonatal Rats with HI-Induced WMD

**Figure 3:**
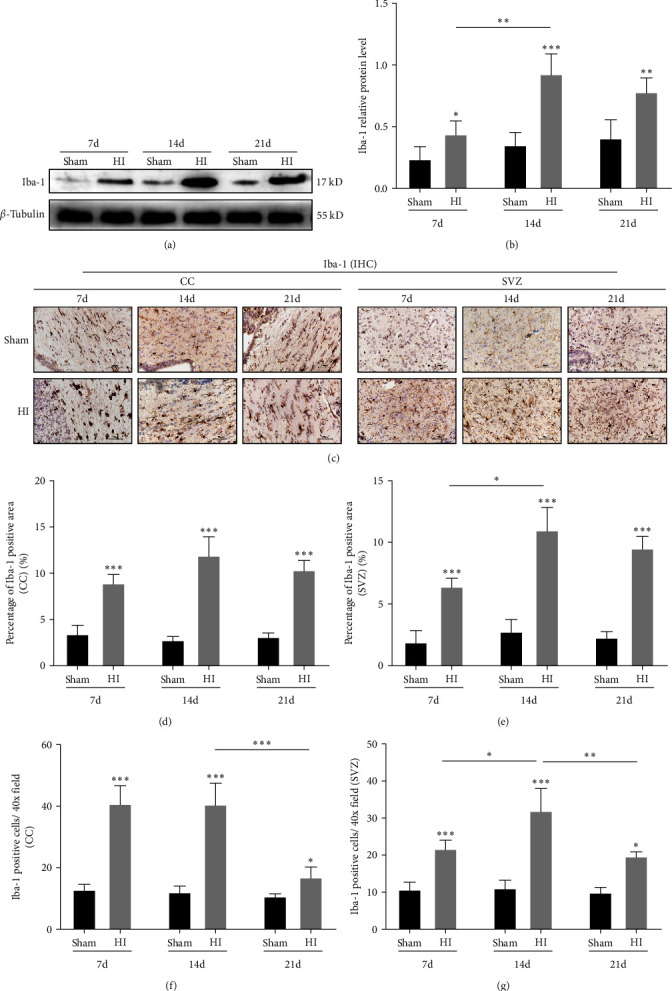
Number of Iba-1-positive microglia changed in the periventricular white matter in neonatal rats with hypoxic–ischemic-induced white matter damage. (a) Western blot detection of Iba-1 levels. (b) Analysis of relative Iba-1 level, with *β*-tubulin used for normalization. (c) IHC staining showing Iba-1 in the corpus callosum (CC) and subventricular zone (SVZ). Scale bar = 50 *μ*m. The percentage of Iba-1-positive area in the (d) CC and (e) SVZ. The Iba-1-positive cells per 40x field in the (f) CC and (g) SVZ. Data are presented as mean ± SEM. Statistical analyses were performed using two-way ANOVA, followed by Tukey's test.  ^*∗*^: Sham vs. HI, or comparison of the same group at different times.  ^*∗*^*P* < 0.05,  ^*∗∗*^*P* < 0.01, and  ^*∗∗∗*^*P* < 0.001. Sham group (*n* = 6); HI group (*n* = 6).

Western blot analysis of the microglia marker Iba-1 showed that Iba-1 protein levels in the periventricular area of the ligated side were significantly higher at 7, 14, and 21 days after HI in the HI group than in the sham group (*P* < 0.05, *P* < 0.01, and *P* < 0.001, respectively; Figures 3(a) and 3(b)). Furthermore, Iba-1 protein levels increased most significantly 14 days after HI in the HI group (*P* < 0.01; Figures 3(a) and 3(b)); however, there were minor differences among the time points in the sham group. The expression of Iba-1 in the CC area and SVZ on the ligated side was analyzed using immunohistochemistry. Both the number of microglia and the area ratio of positive cells increased significantly at 7, 14, and 21 days after HI in the HI group, compared to the sham group (*P* < 0.05, *P* < 0.01, and *P* < 0.001, respectively; Figure 3(c)–3(g)).

**Figure 4:**
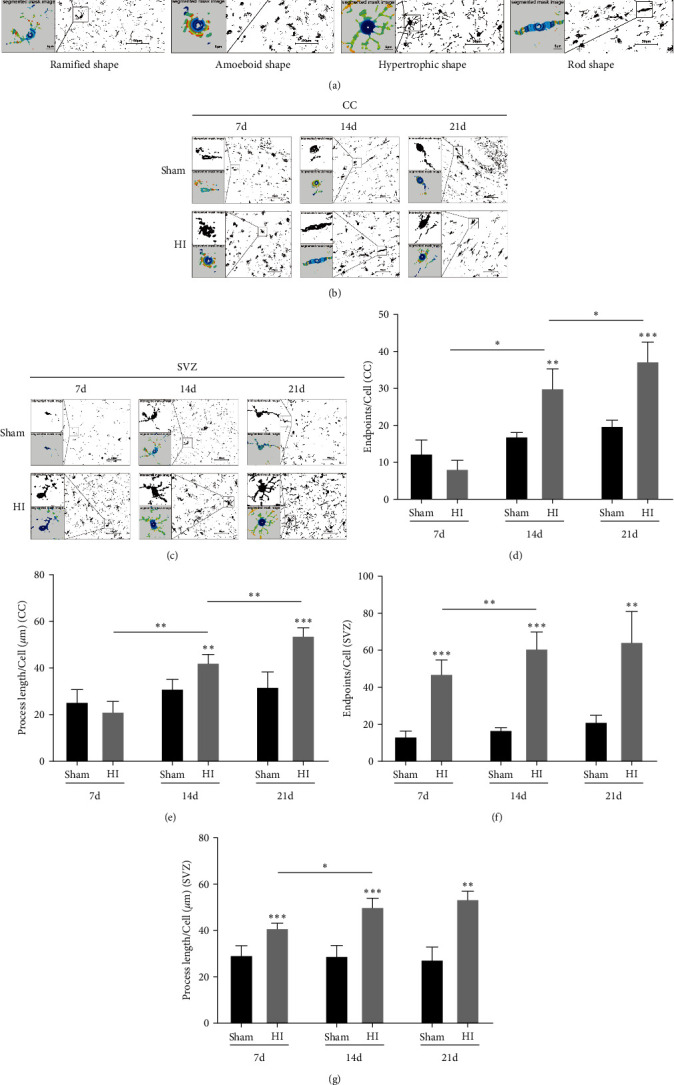
Changes in the morphology of microglial Iba-1 in the periventricular white matter in neonatal rats with hypoxic–ischemic-induced white matter damage. (a) Representative diagram of morphological reconstruction of microglia at different states. Morphological reconstructions of microglia (intersection and segmented mask) in the (b) corpus callosum (CC) and (c) subventricular zone (SVZ). Scale bar = 50 *μ*m. Enlarged scale bar = 5 *μ*m. Endpoints of microglia cells in the (d) CC and (f) SVZ. Process length of microglia cells in the (e) CC and (g) SVZ. Data are presented as mean ± SEM. Statistical analyses were performed using two-way ANOVA, followed by Tukey's test.  ^*∗*^: Sham vs. HI, or comparison of the same group at different times.  ^*∗*^*P* < 0.05,  ^*∗∗*^*P* < 0.01, and  ^*∗∗∗*^*P* < 0.001. Sham group (*n* = 6); HI group (*n* = 6).

Microglia can appear in many forms, usually in bifurcated forms in the resting state [38, 39]. After an injury, microglia can change into amebic, round, hypertrophic, and rod-shaped forms [40] and then secrete inflammatory cytokines. Our microglia morphology analysis revealed that microglia mainly showed branching, small inclusions, and small and long branching processes in the sham group. In the HI group, microglia showed ameba-like morphology when they began to be activated and showed rounder and larger cell bodies and shorter processes than those in the branched forms. After activation, we gradually found more hypertrophic microglia, characterized by enlarged cell bodies, buds, and excessive branching protrusions, rendering their appearance dense, or microglia with a rod-like shape, characterized by a small nucleus, an elongated cell body, and bipolar protrusions in a single direction (Figure 4(a)). In the periventricular area of the CC on the ligated side, microglia in the HI group showed ameba-like morphology 7 days after HI but gradually changed to rod-shaped and hypertrophic morphology 14 and 21 days after HI. The process length and the number of endpoints were significantly higher than those in the sham group (all *P* < 0.001; Figures 4(b), 4(d), and 4(e)). In the SVZ, microglia in the HI group mainly showed fat-like morphology at 7, 14, and 21 days after HI. The process length and the number of endpoints were significantly higher than those in the sham group (all *P* < 0.001; Figures 4(c), 4(f), and 4(g)).

These results showed that from 7 to 21 days after HI-induced brain injury, the expression of Iba-1 in microglia increased, the total number of microglia increased, and their morphology changed, suggesting that the continuous activation of microglia may accompany the development of HI-induced WMD in neonatal rats.

### 3.4. Phenotype Polarization of Microglia in Periventricular White Matter in Neonatal Rats with HI-Induced WMD

**Figure 5:**
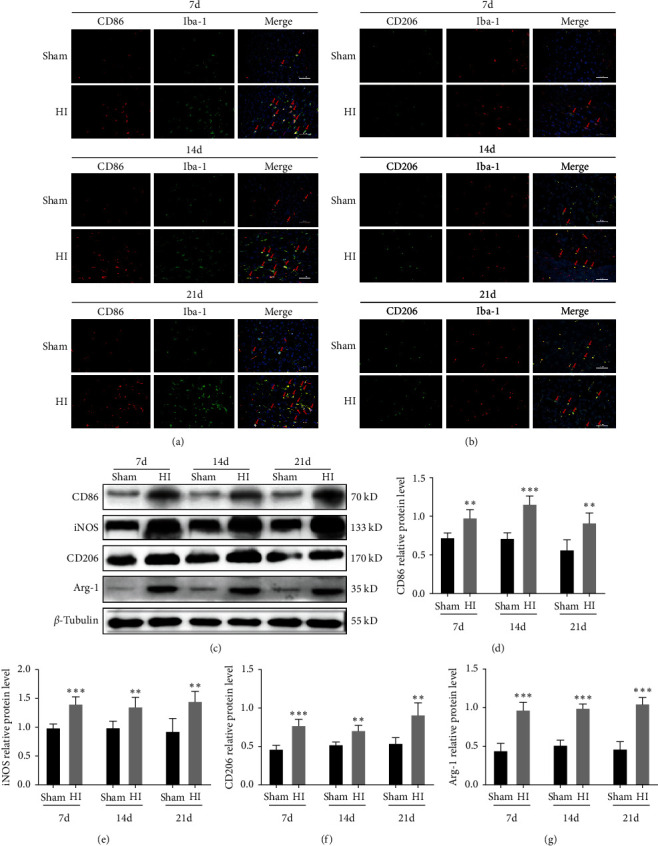
Phenotypic polarization of microglia in the periventricular white matter in neonatal rats with hypoxic–ischemic-induced white matter damage. (a) Representative immunofluorescence images showing colocalization of CD86 (red) and Iba-1 (green) at 7, 14, and 21 days. Scale bar = 50 *μ*m. (b) Representative immunofluorescence images showing colocalization of CD206 (green) and Iba-1 (red) at 7, 14, and 21 days. Scale bar = 50 *μ*m. Sham group (*n* = 6); HI group (*n* = 6). (c) Western blot detection of CD86, CD206, iNOS, and Arg-1 levels. Analyses of relative (d) CD86, (e) iNOS, (f) CD206, and (g) Arg-1 levels, with *β*-tubulin used for normalization. Data represent mean ± SEM. Statistical analyses involved two-way ANOVA, followed by Tukey's test.  ^*∗*^*P* < 0.05,  ^*∗∗*^*P* < 0.01, and  ^*∗∗∗*^*P* < 0.001. Sham group (*n* = 6); HI group (*n* = 6).

Immunofluorescence results showed that there was a greater increase in the number of double-labeled M1 microglia CD86/Iba-1 and M2 microglia CD206/Iba-1 in the periventricular area on the ligated side on Days 7, 14, and 21 after HI in the HI group than in the sham group (Figures 5(a) and 5(b)). In addition, western blot results showed that the protein expression levels of M1 microglia markers, CD86 and iNOS, and M2 microglia markers, CD206 and Arg-1, were significantly upregulated 7, 14, and 21 days after HI in the HI group, compared to the sham group (*P* < 0.01 and *P* < 0.001, respectively; Figure 5(c)–5(g)).

### 3.5. Increased Release of Inflammatory Factors in the Periventricular White Matter in Neonatal Rats with HI-Induced WMD

**Figure 6:**
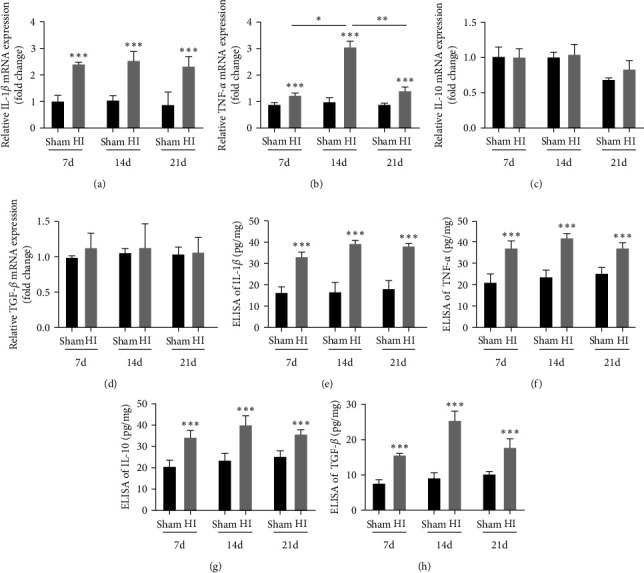
Release of inflammatory factors in the periventricular white matter increased in neonatal rats with hypoxic–ischemic-induced white matter damage. PCR analysis to determine mRNA levels of (a) IL-1*β*, (b) TNF-*α*, (c) IL-10, and (d) TGF-*β*. Levels were normalized against the level of GADPH and expressed as fold change. ELISA to determine levels of (e) IL-1*β*, (f) TNF-*α*, (g) IL-10, and (h) TGF-*β*. Data represent mean ± SEM. Statistical analyses were performed using two-way ANOVA, followed by Tukey's test.  ^*∗*^: Sham vs. HI, or comparison of the same group at different times.  ^*∗*^*P* < 0.05,  ^*∗∗*^*P* < 0.01, and  ^*∗∗∗*^*P* < 0.001. Sham group (*n* = 6); HI group (*n* = 6).

Real-time PCR showed that the expression of M1 microglia-related inflammatory factors (IL-1*β* and TNF-*α*) in the periventricular area on the ligated side was significantly higher at 7, 14, and 21 days after HI in the HI group than in the sham group (*P* < 0.01; Figures 6(a) and 6(b)). However, there was no significant difference in the expression of M2 microglia-related inflammatory factors (IL-10 and TGF-*β*) between the two groups (Figures 6(c) and 6(d)). Therefore, we suggest that HI-induced WMD may promote the transcription of M1 inflammatory factors.

The results of ELISA showed that the levels of M1 factors (IL-1*β* and TNF-*α*) in the tissue homogenate of the periventricular area increased significantly at 7, 14, and 21 days after HI in the HI group, compared to the sham group (all *P* < 0.001; Figures 6(e) and 6(f)) and those of M2 factors (IL-10 and TGF-*β*) also increased significantly at each time point (all *P* < 0.001; Figures 6(g) and 6(h)). This suggests that HI-induced WMD can simultaneously promote the release of IL-1*β* and TNF-*α* by M1 cells and IL-10 and TGF-*β* by M2 cells.

### 3.6. MCC950 Inhibits the Polarization of Microglia into the M1 Phenotype and Improves Brain WMD

**Figure 7:**
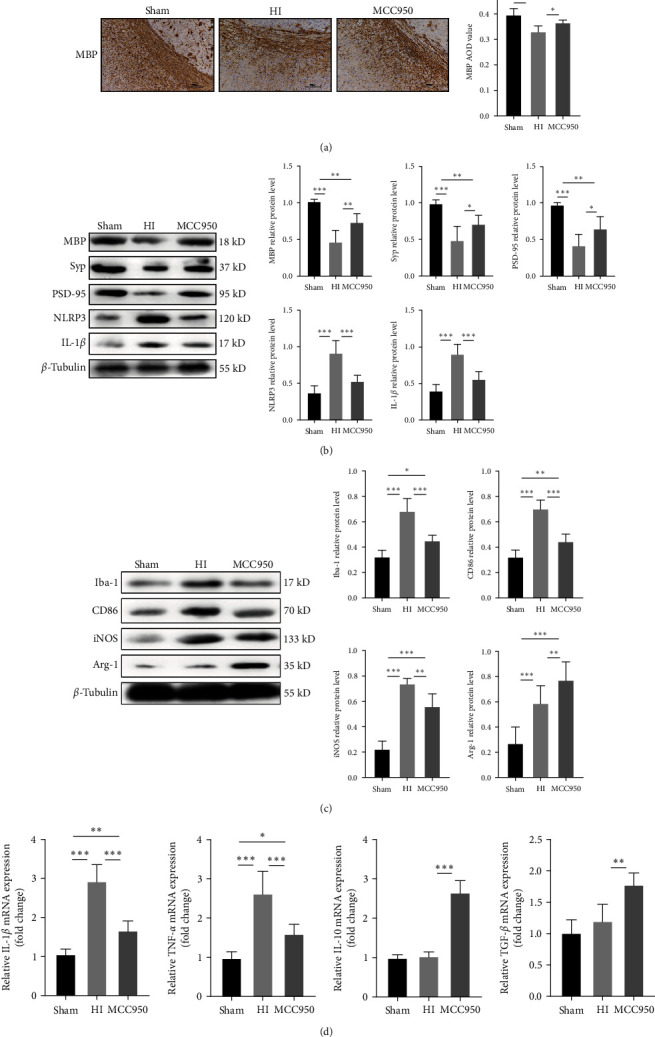
MCC950 inhibits the polarization of microglia into M1 phenotype and improves brain white matter injury. (a) IHC staining showing myelin basic protein (MBP) in the corpus callosum (CC). Scale bar = 100 *μ*m. Western blot detection of (b) MBP, Syp, PSD-95, NLRP3, and IL-1*β*; (c) Iba-1, CD86, iNOS, and Arg-1 levels. (d) PCR analysis to determine mRNA levels of IL-1*β*, TNF-*α*, IL-10, and TGF-*β*. Levels were normalized against the level of GAPDH and expressed as fold change. Data were presented as mean ± SEM. Statistical analyses were performed using one-way ANOVA, followed by Tukey's test.  ^*∗*^*P* < 0.05,  ^*∗∗*^*P* < 0.01, and  ^*∗∗∗*^*P* < 0.001. Sham group (*n* = 6); HI group (*n* = 6); MCC950 group (*n* = 6).

Given the relationship between NLRP3 and neuroinflammation, we evaluated the role of antineuroinflammation in myelin development and synapse formation by inhibiting the NLRP3 inflammasome with MCC950. IHC and western blot analyses showed significantly higher MBP levels 14 days after WMD establishment in the MCC950 group than in the HI group (*P* < 0.05 and *P* < 0.01, respectively; Figures 7(a) and 7(b)). Furthermore, western blot results showed that the levels of Syp and PSD-95 were significantly higher 14 days after WMD establishment in the MCC950 group than in the HI group (all *P* < 0.05; Figure 7(b)).

In addition, western blot results showed that NLRP3 and IL-1*β* levels were lower in the MCC950 group than in the HI group (*P* < 0.001; Figure 7(b)) and that Iba-1, CD86, and iNOS levels were significantly lower in the MCC950 group than in the HI group (*P* < 0.05 and *P* < 0.001, respectively; Figure 7(c)). However, Arg-1 levels were higher in the MCC950 group than in the HI group (*P* < 0.001; Figure 7(c)). To confirm these findings, we performed RT-PCR to detect the expression of cytokines and found that the levels of pro-inflammatory cytokines (IL-1*β* and TNF-*α*) were significantly lower, and anti-inflammatory cytokines (IL-10 and TGF-*β*) were significantly higher in the MCC950 group than in the HI group (*P* < 0.001 and *P* < 0.001, respectively; Figure 7(d)). These results suggest that the inhibition of NLRP3 activation can inhibit the transformation of microglia into the M1 phenotype, promote the release of M2 cytokines, and promote myelin development and synaptic protein formation.

## 4. Discussion

Neonatal HI brain injury mainly causes neuronal injury and WMD, which are the primary causes of death and disability—including cerebral palsy, reduced cognitive function, and continuous motor dysfunction—in preterm and full term infants. Furthermore, HI injury causes selective damage to brain regions based on the time of occurrence and the severity and duration of the injury. Therefore, preterm and full term infants are vulnerable in different brain areas [41, 42]. The pathological features observed in preterm infants are brain atrophy, cerebral WMD, and periventricular leukomalacia, characterized by oligodendrocyte maturation disorder and subsequent myelin dysplasia [41, 43]. In addition, this disorder damages axonal growth, synaptogenesis, and neurogenesis. In this study, using a neonatal rat model of HI-induced cerebral WMD, we found that after HI, the number of oligodendrocytes and the expression of MBP in periventricular white matter decreased, and synaptic formation was impaired, which was consistent with the findings of previous studies [44].

Neuroinflammation is considered an important cause of damage after HI in the immature brain [45]. HI can trigger systemic and central inflammatory responses that can persist for several weeks from the beginning of injury [46, 47]. Studies have shown that HI-induced injuries in the immature brain can cause changes in the expression of immune response and inflammation-related genes, including macrophage and microglia-related genes, T lymphocyte-related genes, and cytokines [9]. Simultaneously, innate and acquired immune responses can be observed 7 days after HI-induced cerebral WMD, characterized by the imbalance of Th1/Th17 and Th2/Treg, which is accompanied by significant increases in the levels of M1 Th1/Th17-related transcription factors and cytokines, whereas the levels of Th2/Treg-related transcription factors and cytokines are reduced or remain unchanged [48]. Notably, when an autopsy on the brain tissue of infants with HI brain injury was performed, clinical studies found that the chronic upregulation of cytokine expression and gliosis are closely associated with poor neural development [49, 50]. These results indicate that neuroinflammation plays a key pathogenic role in HI-induced brain injury.

Activation of the NLRP3 inflammasome is an important neuroinflammation marker [15], and its activation is associated with the occurrence and development of various nervous system diseases. It is also considered a main driving factor of neuroinflammation and an underlying cause of neurobehavioral disorders [31]. After activation, the NLRP3 inflammasome associates with scaffold proteins, apoptosis-associated speck-like protein containing a caspase-1 recruitment domain (ASC), and pro-caspase-1 to mediate IL-1*β* and IL-18 release [51]. Studies have shown that the level of NLRP3 is upregulated after neonatal HI and is expressed in astrocytes during the early stage (3 hr), and it is widely expressed in microglia after 24–72 hr [52]. After HI brain injury in neonatal rats, caspase-1 cleavage, and IL-1 levels can be reduced by inhibiting the downregulation of thioredoxin interacting protein (TXNIP) *β* expression. The decreased production of NLRP3 inhibits the activation of NLRP3 inflammatory bodies, thereby reducing the volume of cerebral infarction [53]. Recent studies have shown that NLRP3 inflammatory corpuscles are key pathogenic effectors that lead to stroke-induced destruction of the blood–brain barrier by activating the inflammatory signaling cascade leading to endothelial cell death in the brain [54]. The NLRP3 inflammasome pathway and complex formation contribute to the activation of inflammatory caspases and release of inflammatory cytokines, including IL-1*β*, TNF- *α*, IL-18, and vascular endothelial growth factor [55]. In this study, the expression of the NLRP3 inflammasome and its related proteins (caspase-1 and IL-1*β*) was higher in the HI group than in the sham group. At 7 days after HI injury, the expression level of NLRP3 inflammasomes in the periventricular area was higher in the HI group than in the sham group. At 7, 14, and 21 days after HI, the levels of NLRP3 inflammasomes and its activation-related proteins (caspase-1 and IL-1*β*) were also higher in the HI group than in the sham group, suggesting that there may be continuous NLRP3 inflammasome activation in HI-induced WMD.

Microglia are innate immune cells of the central nervous system (CNS) and the main mediator of neuroinflammation. They account for 10%–20% of all glial cells. Microglia originate from the embryo's yolk sac, migrate to the CNS in the early stages of development, and remain stable throughout adulthood [56, 57]. During development, microglia play an important role in maintaining the normal function of the CNS, including synaptic pruning, control of axonal growth, oligodendrocyte differentiation, phagocytosis, and the removal of cell debris, particularly for the maintenance of myelin homeostasis [58–60]. After infection or inflammation, microglia in the brain lose their normal steady-state function, become active, trigger neuroinflammation, and then accelerate the development of brain injury in preterm infants. Clinical studies have revealed that the number of activated microglia is significantly increased in the brain tissue of children with WMD [61]. HI may activate the JAK2/STAT3 signaling pathway, leading to microglial activation and neuroinflammation [62]. Perinatal HI can activate the immune system and trigger peripheral and central responses, which involve immune cell activation, an increase in the production of immune mediators, and the release of reactive oxygen species (ROS) [63]. Similarly, microglia activated in vitro can impair the maturation of oligodendrocytes by excessively releasing M1 cytokines [64], free radicals, such as ROS and nitric oxide synthase [64, 65], and excitotoxic molecules (such as glutamate) [66, 67]. M1 microglia subsets secrete cytokines and ROS, which can directly damage oligodendrocytes and lead to demyelination. M2 microglia subsets secrete nutritional factors and promote oligodendrocyte migration and differentiation, resulting in myelin sheath reformation [68]. In addition, microglia can also secrete various synaptic signaling molecules required to maintain normal synaptic function. For example, thrombin reactive protein can promote axon growth and induce synaptic formation [69]. Transmembrane polypeptide KARAP/DAP12 can alter synaptic function and plasticity by changing the physiological function of microglia [70]. Mice lacking the CX3C chemokine receptor 1 expressed by microglia can exhibit defects in synaptic pruning [71]. Neurons exposed to a culture medium of activated microglia demonstrate severe synaptic loss, DNA breakage, and neuronal cell death [72]. Therefore, determining the regional and temporal heterogeneity and the specific role of microglia in HI brain injury is of great significance for elucidating the mechanism of HI brain injury.

In this study, the expression region and microglia morphology were analyzed to evaluate the activation process of microglia after HI injury. We found that microglia in the CC area of rats in the HI group and the sham group displayed ameba-like morphology 7 days after HI; however, the number of microglia was higher in the HI group than in the sham group. From 14 to 21 days after HI, the morphology of microglia in the HI and sham groups changed. Microglia in the sham group were mainly ramified, whereas those in the HI group were mostly hypertrophic or rod shaped. Furthermore, we found that from 7 to 21 days after HI, the microglia in the SVZ were mainly ramified in the sham group, whereas those in the HI group were mostly hypertrophic. These changes provide strong evidence of microglial activation after HI injury. In addition, the morphology of microglia can change during the developmental process. Microglia before and after birth demonstrate an ameba-like morphology, transitioning to a branched morphology in the 2nd week after birth [73, 74]. Amoebic microglia have been found in almost all brain regions from postnatal Day 0 (P0) to postnatal Day 20 (P20); however, they are more prominent in the CC [75]. They are more specifically concentrated in the supraventricular CC, subventricular area of the lateral ventricle, and transparent septum [76]. This can also explain the existence of amebic microglia in the CC 7 days after HI in the sham and HI groups, which may be associated with myelination. With increasing age, microglia branching increased in the CC, and the amebic changes decreased, suggesting that microglia tend to mature gradually approximately 2 weeks after birth. However, the time and order of changes in microglial morphology in each region of brain tissue after HI need to be further investigated.

Microglia are a heterogeneous population and their members are distinguished according to their functional abilities [77, 78]. Activated microglia can exhibit various phenotypes and play different pathogenic and protective/regenerative roles. According to previous research, activated microglia can be roughly divided into different polarization states: activated M1 polarization (cytotoxic, often associated with the M1 response) or activated M2a polarization (associated with the M2 response or tissue repair), and acquired inactivated M2b polarization (associated with immunosuppression and regulation) [79, 80]. Microglia in an inactive state are called steady-state microglia (M0). The M1 type expresses markers such as CD16/32 and CD68 and releases destructive mediators such as TNF-*α*, iNOS, and IL-1*β*. Conversely, the M2 type expresses markers such as Arg-1, YM-1, and CD206, and produces beneficial mediators, including IL-4, IL-10, and TGF-*β*, which promote tissue repair and support neuronal survival [81].

The interaction between M1/M2 microglia after neonatal HI brain injury appears to be a complex, time-dependent continuum with an early M1 response and delayed M2 response, characterized by the expression of surface receptors and phenotypic-specific mediators [82]. Over time, microglia release neurotrophic factors, such as brain-derived neurotrophic factor, IL-10, IL-4, TGF-*β*, IL-13, and IGF-1, which play a protective role (M2 type) [83]. In addition, after neonatal HI injury, the number of M2 microglia changes with M1 activation and apoptosis [84].

In this study, we observed that from 7 to 21 days after HI, there was an increase in the levels of the microglia M1 phenotypic marker CD86/Iba-1 and microglia M2 phenotypic marker CD206/Iba-1 in the periventricular region. This result indicates that the phenotypic polarization of microglia is a continuous process accompanied by the development of HI-induced WMD in the brain. Furthermore, both PCR and ELISA results confirmed that on Days 7, 14, and 21 after HI, the pro-inflammatory factors produced by M1 in the HI group increased compared to the sham group in both transcription and secretion levels, while the anti-inflammatory factors produced by M2 increased in secretion levels, compared to the sham group; however, there was no significant difference in transcription levels between the two groups. It is considered that HI may inhibit the transcription of M2-inducing cytokines. However, the posttranslational modification and function of its protein may increase. This may be related to the activation of the M2 phenotype by hypoxia and ischemia, enhancing its protein self-repair function. Therefore, in the future, the potential mechanism of functional transformation of microglia after hypoxic brain damage can be further determined by detecting the protein expression, transcription level and secretion level of cytokines at consecutive time points.

MCC950 is a small molecular compound similar to sulfonylurea, which has been shown to block NLRP3-induced ASC oligomerization, and it is an efficient and highly selective NLRP3 inhibitor [33, 34]. According to previous reports, MCC950 treatment inhibits the activation of NLRP3 and reduces the level of IL-1 in the body. The application of MCC950 decreased the severity of experimental autoimmune encephalomyelitis (EAE) [34]. MCC950 treatment can enhance the effect of microglia on amyloid beta accumulation and improve the cognitive deficit in mouse models of Alzheimer's Disease, which is related to inhibiting the activation of inflammatory bodies and microglia [85]. Pieces of evidence have shown that the selective NLRP3 inflammasome inhibitor can effectively reduce the expression of pro-inflammatory cytokines and increase the expression of anti-inflammatory cytokines [26]. In this study, we investigated the protective effect of MCC950 in a neonatal rat model of HI-induced WMD. Our results indicate that MCC950 treatment inhibits the activation of NLRP3 inflammasomes and increases synaptic protein damage around 14 days after HI brain damage. In addition, we found that MCC950 treatment can inhibit the transformation of microglia into the M1 phenotype, promote the transformation of the M2 phenotype, reduce proinflammatory cytokines, and increase anti-inflammatory factors. These results also indicate that the NLRP3 inflammasome may play a role in inflammatory damage caused by HI-induced WMD. MCC950 treatment may be a potential candidate for the treatment of WMD.

## 5. Conclusions

Inhibition of NLRP3 can play a role in transforming the M2 microglial phenotype to reduce neuroinflammation by inhibiting the differentiation of microglia into the M1 phenotype, thereby improving myelin development and synapse formation. Microglia are key mediators of the inflammatory response after HI-induced WMD and exhibit continuous phenotypic polarization 7–21 days after HI-induced WMD. This finding can potentially lead to a new treatment regimen targeting the phenotypic polarization of microglia early after HI brain injury. However, this article has some limitations. First of all, the mechanism of the difference in transcription and protein expression levels of M1 and M2 factors and the specific time point are not yet clear. Further data about protein expression and transcription and secretion levels of cytokines at continuous time points are required to determine the potential mechanisms of microglial functional transformations after HI brain injury. Second, MCC950 regulates the polarization imbalance of microglia, which is the key way to exert the brain protection, and may be related to the inhibition of the NLRP3 inflammasome. In our subsequent research, its role still needs to be explored to elucidate the overall mechanism of the neuroprotective effect of MCC950. Finally, white matter injury in premature infants caused by HI mainly affects learning and cognition, as proved by our previous studies. However, we have not made corresponding evaluations of the occurrence of epilepsy and long-term dysplasia. We will explore these matters in future experiments.

## Data Availability

The data used to support the findings of this study are available from the corresponding author upon request.
